# Supplementary material for: Effects of harvest number on the yield and quality of different alfalfa varieties under hydroponic conditions
Source: PLoS One. 2026 Apr 16;21(4):e0346431. doi: 10.1371/journal.pone.0346431 (PMC13086426; doi:10.1371/journal.pone.0346431)
Supplement: S1 Table — (PDF) [file pone.0346431.s001.pdf]

Variety	Cuts	ADF/DM	AVERAGE	STDEVP	NDF/DM
Zhongcao No. 13-①	1	35.37			38.41
Zhongcao No. 13-②	1	35.51	35.44	0.06	38.59
Zhongcao No. 13-③	1	35.45			38.49
WL440HQ-①	1	27.16			31.08
WL440HQ-②	1	27.14	27.15	0.01	31.17
WL440HQ-③	1	27.16			31.13
WL525HQ-①	1	28.65			31.79
WL525HQ-②	1	28.60	28.62	0.02	31.27
WL525HQ-③	1	28.62			31.53
WL903-①	1	25.74			32.23
WL903-②	1	25.77	25.75	0.01	32.63
WL903-③	1	25.74			32.44
WL712-①	1	25.81			31.79
WL712-②	1	26.75	26.28	0.38	31.89
WL712-③	1	26.27			31.85
Zhongcao No. 13-①	2	34.99			37.70
Zhongcao No. 13-②	2	34.48	34.74	0.21	37.49
Zhongcao No. 13-③	2	34.73			37.59
WL440HQ-①	2	26.04			30.36
WL440HQ-②	2	26.40	26.22	0.15	30.59
WL440HQ-③	2	26.22			30.47
WL525HQ-①	2	29.39			31.79
WL525HQ-②	2	29.12	29.26	0.11	31.51
WL525HQ-③	2	29.27			31.64
WL903-①	2	24.85			30.47
WL903-②	2	24.90	24.88	0.02	30.73
WL903-③	2	24.89			30.60
WL712-①	2	25.86			30.85
WL712-②	2	26.00	25.93	0.06	31.00
WL712-③	2	25.92			30.91
Zhongcao No. 13-①	3	34.20			37.45
Zhongcao No. 13-②	3	34.24	34.22	0.02	37.21
Zhongcao No. 13-③	3	34.21			37.33
WL440HQ-①	3	26.39			30.83
WL440HQ-②	3	26.58	26.49	0.08	30.88
WL440HQ-③	3	26.50			30.88
WL525HQ-①	3	28.39			31.03
WL525HQ-②	3	28.19	28.29	0.08	30.54
WL525HQ-③	3	28.27			30.80
WL903-①	3	25.43			30.03
WL903-②	3	25.05	25.24	0.15	30.21
WL903-③	3	25.23			30.12
WL712-①	3	26.00			30.75
WL712-②	3	25.55	25.77	0.18	30.62
WL712-③	3	25.76			30.68
Zhongcao No. 13-①	4	34.69			36.81
Zhongcao No. 13-②	4	34.62	34.66	0.03	37.46
Zhongcao No. 13-③	4	34.66			37.14
WL440HQ-①	4	27.05			31.41
WL440HQ-②	4	26.93	26.99	0.05	30.93

WL440HQ-③	4	26.99			31.16
WL525HQ-①	4	26.01			30.74
WL525HQ-②	4	26.28	26.15	0.11	30.89
WL525HQ-③	4	26.15			30.81
WL903-①	4	24.27			29.10
WL903-②	4	24.45	24.36	0.07	29.02
WL903-③	4	24.36			29.06
WL712-①	4	25.70			31.24
WL712-②	4	26.33	26.02	0.26	31.68
WL712-③	4	26.03			31.48
Zhongcao No. 13-①	5	33.03			36.38
Zhongcao No. 13-②	5	33.42	33.23	0.16	36.24
Zhongcao No. 13-③	5	33.22			36.29
WL440HQ-①	5	24.98			29.37
WL440HQ-②	5	24.84	24.91	0.06	29.86
WL440HQ-③	5	24.91			29.61
WL525HQ-①	5	28.11			31.01
WL525HQ-②	5	28.05	28.08	0.03	31.01
WL525HQ-③	5	28.09			31.01
WL903-①	5	23.39			29.12
WL903-②	5	23.02	23.21	0.15	28.88
WL903-③	5	23.22			29.00
WL712-①	5	25.83			31.29
WL712-②	5	26.39	26.11	0.23	32.66
WL712-③	5	26.12			31.96
Zhongcao No. 13-①	6	29.36			35.53
Zhongcao No. 13-②	6	29.59	29.47	0.09	35.97
Zhongcao No. 13-③	6	29.47			35.74
WL440HQ-①	6	24.87			29.06
WL440HQ-②	6	24.28	24.58	0.24	29.12
WL440HQ-③	6	24.59			29.08
WL525HQ-①	6	25.94			29.88
WL525HQ-②	6	26.17	26.05	0.09	29.73
WL525HQ-③	6	26.05			29.80
WL903-①	6	22.74			28.44
WL903-②	6	22.80	22.77	0.03	28.18
WL903-③	6	22.76			28.32
WL712-①	6	24.49			29.41
WL712-②	6	24.36	24.42	0.05	29.64
WL712-③	6	24.42			29.52

AVERAGE	STDEVP	DMI/DM	AVERAGE	STDEVP	DDM/DM
		3.12			61.35
38.50	0.07	3.11	3.12	0.01	61.24
		3.12			61.29
		3.86			67.74
31.13	0.04	3.85	3.86	0.00	67.76
		3.86			67.74
		3.77			66.58
31.53	0.21	3.84	3.81	0.03	66.62
		3.81			66.61
		3.72			68.85
32.43	0.17	3.68	3.70	0.02	68.83
		3.70			68.85
		3.78			68.79
31.84	0.04	3.76	3.77	0.01	68.07
		3.77			68.43
		3.18			61.64
37.60	0.09	3.20	3.19	0.01	62.04
		3.19			61.84
		3.95			68.62
30.47	0.09	3.92	3.94	0.01	68.34
		3.94			68.48
		3.77			66.00
31.65	0.11	3.81	3.79	0.01	66.22
		3.79			66.10
		3.94			69.54
30.60	0.11	3.91	3.92	0.01	69.50
		3.92			69.51
		3.89			68.75
30.92	0.06	3.87	3.88	0.01	68.64
		3.88			68.71
		3.20			62.26
37.33	0.10	3.22	3.21	0.01	62.23
		3.21			62.25
		3.89			68.34
30.86	0.02	3.89	3.89	0.00	68.19
		3.89			68.26
		3.87			66.78
30.79	0.20	3.93	3.90	0.02	66.94
		3.90			66.87
		4.00			69.09
30.12	0.07	3.97	3.98	0.01	69.39
		3.98			69.24
		3.90			68.65
30.69	0.05	3.92	3.91	0.01	69.00
		3.91			68.84
		3.26			61.87
37.14	0.26	3.20	3.23	0.02	61.93
		3.23			61.90
		3.82			67.83
31.17	0.19	3.88	3.85	0.02	67.92

		3.85			67.87
		3.90			68.64
30.81	0.06	3.88	3.89	0.01	68.43
		3.90			68.53
		4.12			70.00
29.06	0.03	4.14	4.13	0.00	69.85
		4.13			69.93
		3.84			68.88
31.47	0.18	3.79	3.81	0.02	68.39
		3.81			68.62
		3.30			63.17
36.31	0.06	3.31	3.31	0.01	62.87
		3.31			63.02
		4.09			69.44
29.61	0.20	4.02	4.05	0.03	69.55
		4.05			69.50
		3.87			67.00
31.01	0.00	3.87	3.87	0.00	67.05
		3.87			67.02
		4.12			70.68
29.00	0.10	4.16	4.14	0.01	70.97
		4.14			70.81
		3.84			68.78
31.97	0.56	3.67	3.75	0.07	68.34
		3.75			68.55
		3.38			66.03
35.75	0.18	3.34	3.36	0.02	65.85
		3.36			65.94
		4.13			69.52
29.09	0.02	4.12	4.13	0.00	69.99
		4.13			69.75
		4.02			68.70
29.80	0.06	4.04	4.03	0.01	68.51
		4.03			68.61
		4.22			71.18
28.31	0.11	4.26	4.24	0.02	71.14
		4.24			71.17
		4.08			69.82
29.53	0.09	4.05	4.06	0.01	69.93
		4.06			69.88

AVERAGE	STDEVP	RFV/DM	AVERAGE	STDEVP	CP/DM
		148.57			25.92
61.29	0.05	147.61	148.10	0.40	25.78
		148.13			25.85
		202.77			33.55
67.75	0.01	202.20	202.47	0.23	33.27
		202.44			33.42
		194.79			31.95
66.60	0.02	198.20	196.51	1.39	31.83
		196.53			31.89
		198.74			28.13
68.84	0.01	196.22	197.46	1.03	28.04
		197.43			28.09
		201.32			33.47
68.43	0.30	198.53	199.91	1.14	33.45
		199.89			33.45
		152.08			22.69
61.84	0.16	153.92	153.01	0.75	22.82
		153.02			22.76
		210.27			32.54
68.48	0.11	207.84	209.06	0.99	32.59
		209.07			32.58
		193.12			34.09
66.11	0.09	195.47	194.30	0.96	34.04
		194.33			34.05
		212.33			30.41
69.52	0.02	210.40	211.34	0.79	30.57
		211.29			30.49
		207.30			32.91
68.70	0.04	206.00	206.70	0.53	32.88
		206.80			32.89
		154.66			22.63
62.25	0.01	155.55	155.11	0.36	22.26
		155.13			22.04
		206.17			34.49
68.26	0.06	205.45	205.75	0.30	34.34
		205.64			34.46
		200.23			33.88
66.86	0.06	203.86	202.02	1.48	33.26
		201.98			33.37
		214.04			31.26
69.24	0.12	213.65	213.84	0.16	31.25
		213.85			31.27
		207.64			32.22
68.83	0.14	209.58	208.64	0.79	32.57
		208.69			32.40
		156.34			23.02
61.90	0.03	153.79	155.05	1.04	22.12
		155.02			22.36
		200.89			34.99
67.88	0.04	204.24	202.59	1.37	35.36

		202.63			35.47
		207.73			32.37
68.53	0.09	206.07	206.90	0.68	32.60
		206.91			32.34
		223.79			30.68
69.93	0.06	223.95	223.86	0.07	30.69
		223.84			30.63
		205.08			31.20
68.63	0.20	200.79	202.89	1.75	31.27
		202.78			31.28
		161.50			23.54
63.02	0.12	161.35	161.46	0.07	24.02
		161.52			24.06
		219.92			33.72
69.50	0.05	216.71	218.31	1.31	33.68
		218.31			33.67
		200.98			32.13
67.02	0.02	201.15	201.05	0.07	31.95
		201.03			32.00
		225.81			29.29
70.82	0.12	228.62	227.19	1.15	29.49
		227.14			29.37
		204.49			31.45
68.56	0.18	194.67	199.55	4.01	31.85
		199.50			31.59
		172.88			25.66
65.94	0.07	170.28	171.59	1.06	25.89
		171.62			25.89
		222.55			32.52
69.75	0.19	223.60	223.07	0.43	32.37
		223.07			32.49
		213.89			34.29
68.61	0.07	214.39	214.16	0.21	34.26
		214.19			34.28
		232.86			31.28
71.16	0.02	234.84	233.84	0.81	31.06
		233.81			31.01
		220.81			33.76
69.88	0.04	219.43	220.15	0.57	33.48
		220.20			33.40

AVERAGE	STDEVP	Hay yield Kg/m^2	AVERAGE	STDEVP	leaf-stem ratio
		0.38			0.56
25.85	0.06	0.38	0.38	0.00	0.55
		0.38			0.57
		0.47			0.78
33.41	0.11	0.47	0.47	0.00	0.78
		0.48			0.79
		0.15			0.78
31.89	0.05	0.15	0.15	0.00	0.80
		0.15			0.83
		0.54			0.85
28.09	0.03	0.53	0.54	0.00	0.83
		0.54			0.85
		0.22			0.82
33.46	0.01	0.22	0.22	0.00	0.83
		0.22			0.84
		0.28			0.71
22.76	0.05	0.28	0.28	0.00	0.71
		0.28			0.70
		0.31			0.84
32.57	0.02	0.31	0.31	0.00	0.77
		0.32			0.78
		0.12			0.90
34.06	0.02	0.12	0.12	0.00	0.90
		0.12			0.90
		0.58			0.90
30.49	0.07	0.58	0.58	0.01	0.92
		0.60			0.91
		0.27			1.19
32.90	0.01	0.26	0.26	0.00	1.13
		0.26			1.18
		0.24			0.73
22.31	0.24	0.24	0.24	0.00	0.72
		0.23			0.74
		0.37			0.90
34.43	0.07	0.37	0.37	0.00	0.87
		0.37			0.90
		0.11			0.94
33.51	0.27	0.11	0.11	0.00	0.95
		0.12			0.94
		0.43			0.79
31.26	0.01	0.44	0.44	0.01	0.79
		0.45			0.79
		0.30			1.03
32.40	0.14	0.29	0.30	0.00	1.07
		0.30			1.05
		0.27			0.72
22.50	0.38	0.27	0.27	0.00	0.72
		0.27			0.72
		0.31			0.80
35.27	0.21	0.32	0.32	0.00	0.81

		0.31			0.79
		0.16			0.93
32.44	0.11	0.16	0.16	0.00	0.95
		0.16			0.94
		0.40			1.00
30.67	0.03	0.41	0.41	0.00	1.00
		0.41			1.02
		0.26			1.15
31.25	0.04	0.26	0.26	0.00	1.15
		0.26			1.11
		0.32			0.72
23.87	0.24	0.31	0.31	0.00	0.70
		0.31			0.73
		0.33			1.09
33.69	0.02	0.33	0.33	0.00	1.07
		0.33			1.07
		0.19			1.13
32.03	0.08	0.19	0.19	0.00	1.17
		0.19			1.16
		0.56			0.97
29.38	0.08	0.58	0.57	0.01	1.00
		0.57			0.98
		0.33			1.15
31.63	0.17	0.33	0.33	0.00	1.15
		0.32			1.13
		0.30			0.70
25.81	0.11	0.30	0.30	0.00	0.67
		0.30			0.70
		0.43			1.09
32.46	0.07	0.42	0.42	0.00	1.05
		0.42			1.07
		0.22			1.05
34.28	0.01	0.22	0.22	0.00	1.08
		0.22			1.04
		0.41			0.80
31.12	0.12	0.41	0.41	0.00	0.83
		0.41			0.82
		0.39			1.03
33.55	0.15	0.39	0.39	0.00	1.03
		0.39			1.04

AVERAGE	STDEVP	DM%	AVERAGE	STDEVP
		0.18		
0.56	0.01	0.18	0.18	0.00
		0.18		
		0.16		
0.78	0.01	0.15	0.16	0.00
		0.16		
		0.16		
0.80	0.02	0.16	0.16	0.00
		0.16		
		0.16		
0.84	0.01	0.16	0.16	0.00
		0.16		
		0.14		
0.83	0.01	0.14	0.14	0.00
		0.14		
		0.18		
0.71	0.00	0.18	0.18	0.00
		0.17		
		0.16		
0.80	0.03	0.16	0.16	0.00
		0.16		
		0.16		
0.90	0.00	0.16	0.16	0.00
		0.16		
		0.16		
0.91	0.01	0.16	0.16	0.00
		0.17		
		0.14		
1.16	0.03	0.14	0.14	0.00
		0.14		
		0.18		
0.73	0.01	0.18	0.18	0.00
		0.17		
		0.16		
0.89	0.01	0.16	0.16	0.00
		0.16		
		0.16		
0.94	0.00	0.15	0.16	0.00
		0.16		
		0.16		
0.79	0.00	0.16	0.16	0.00
		0.17		
		0.14		
1.05	0.02	0.14	0.14	0.00
		0.14		
		0.18		
0.72	0.00	0.18	0.18	0.00
		0.18		
		0.16		
0.80	0.01	0.16	0.16	0.00

		0.16		
		0.15		
0.94	0.01	0.16	0.16	0.00
		0.16		
		0.16		
1.00	0.01	0.16	0.16	0.00
		0.17		
		0.14		
1.13	0.02	0.14	0.14	0.00
		0.14		
		0.18		
0.71	0.01	0.18	0.18	0.00
		0.17		
		0.16		
1.08	0.01	0.16	0.16	0.00
		0.15		
		0.16		
1.15	0.02	0.16	0.16	0.00
		0.15		
		0.16		
0.98	0.02	0.16	0.16	0.00
		0.16		
		0.14		
1.15	0.01	0.14	0.14	0.00
		0.14		
		0.18		
0.69	0.01	0.18	0.18	0.00
		0.18		
		0.16		
1.07	0.01	0.15	0.16	0.00
		0.15		
		0.16		
1.06	0.01	0.16	0.16	0.00
		0.16		
		0.16		
0.81	0.01	0.16	0.16	0.00
		0.16		
		0.14		
1.03	0.00	0.14	0.14	0.00
		0.14		
