# Supplementary figures and images for: Effects of harvest number on the yield and quality of different alfalfa varieties under hydroponic conditions
Source: PLoS One. 2026 Apr 16;21(4):e0346431. doi: 10.1371/journal.pone.0346431 (PMC13086426; doi:10.1371/journal.pone.0346431)

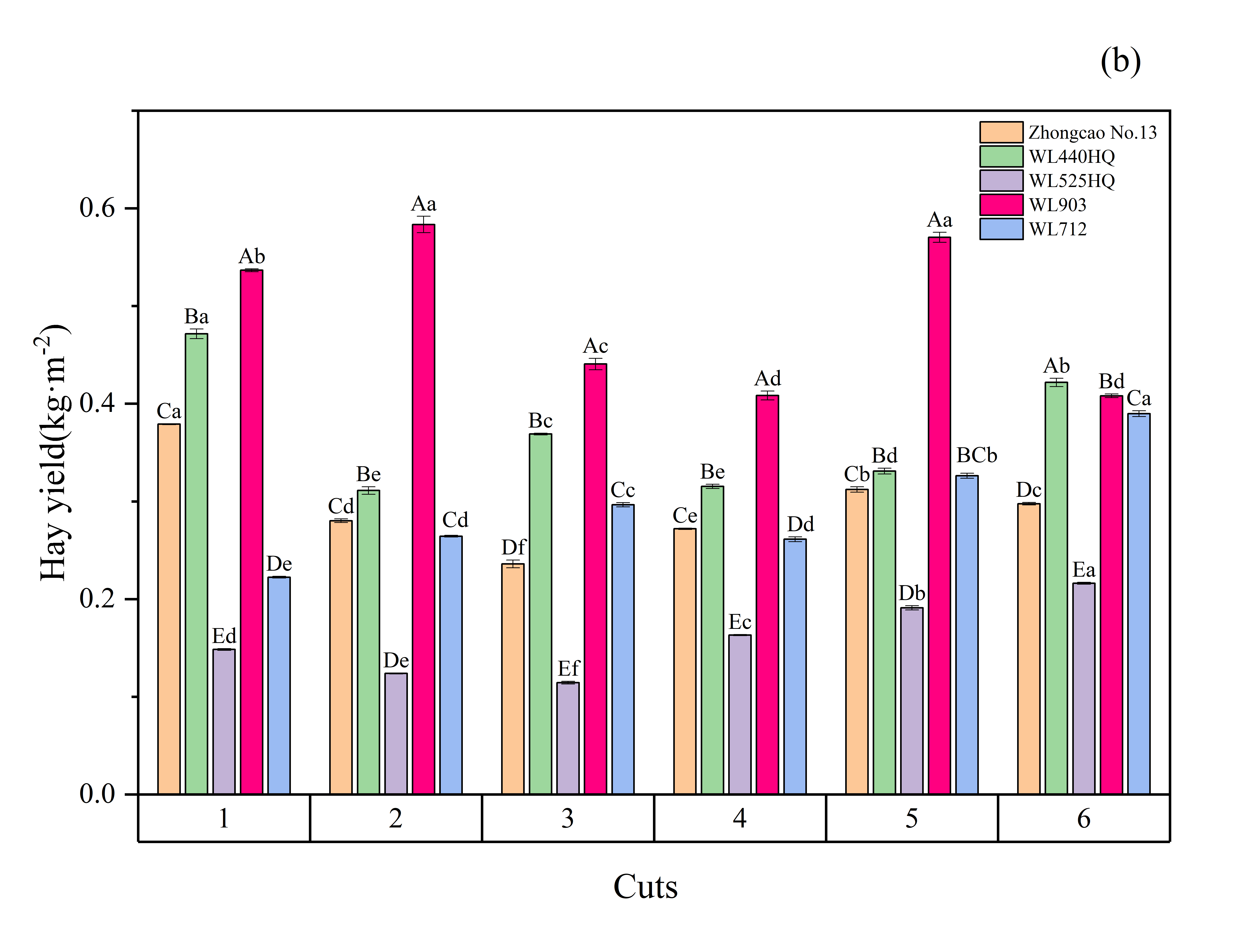

Supplement: S1 Fig — (TIF) [file pone.0346431.s002.tif]

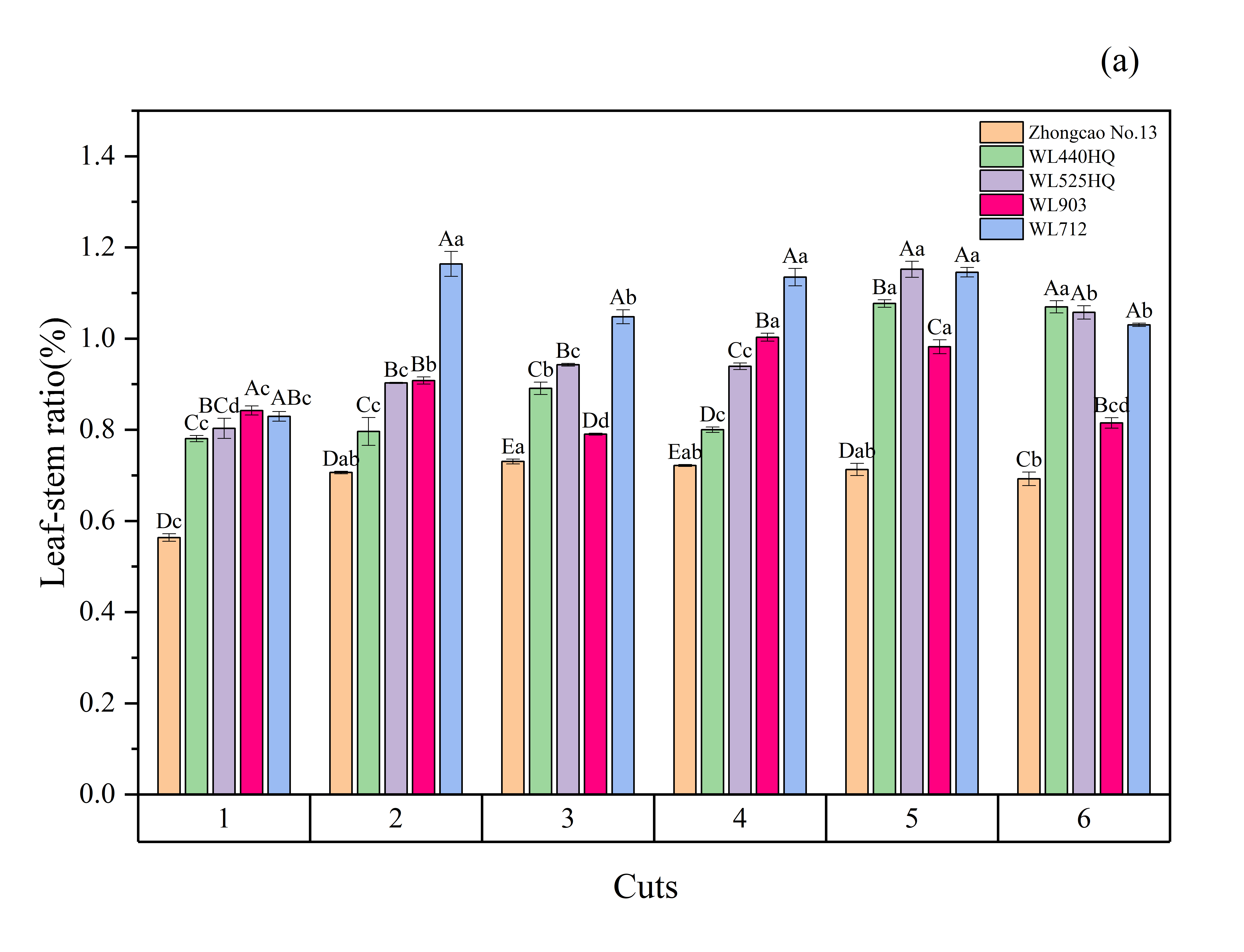

Supplement: S2 Fig — (TIF) [file pone.0346431.s003.tif]

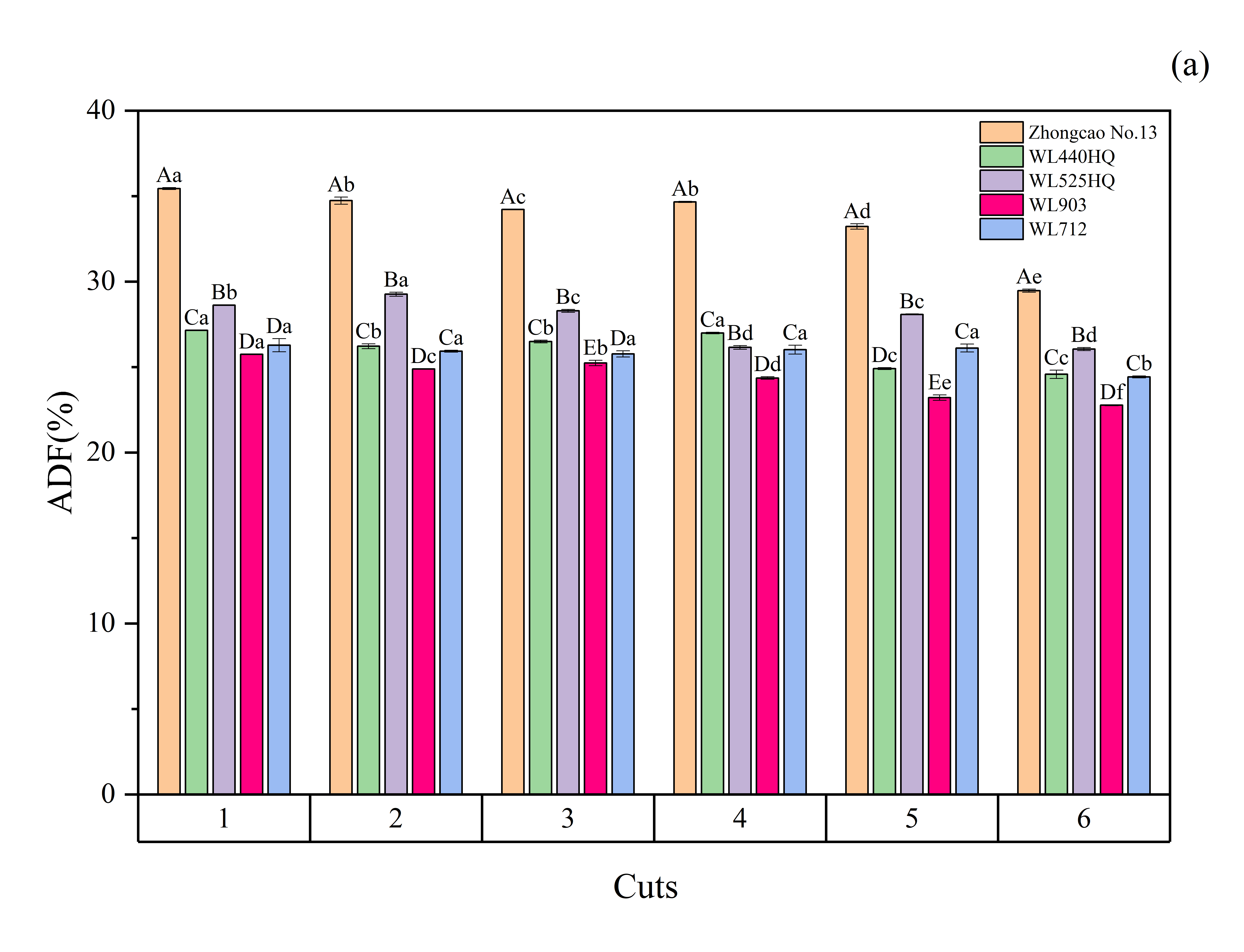

Supplement: S3 Fig — (TIF) [file pone.0346431.s004.tif]

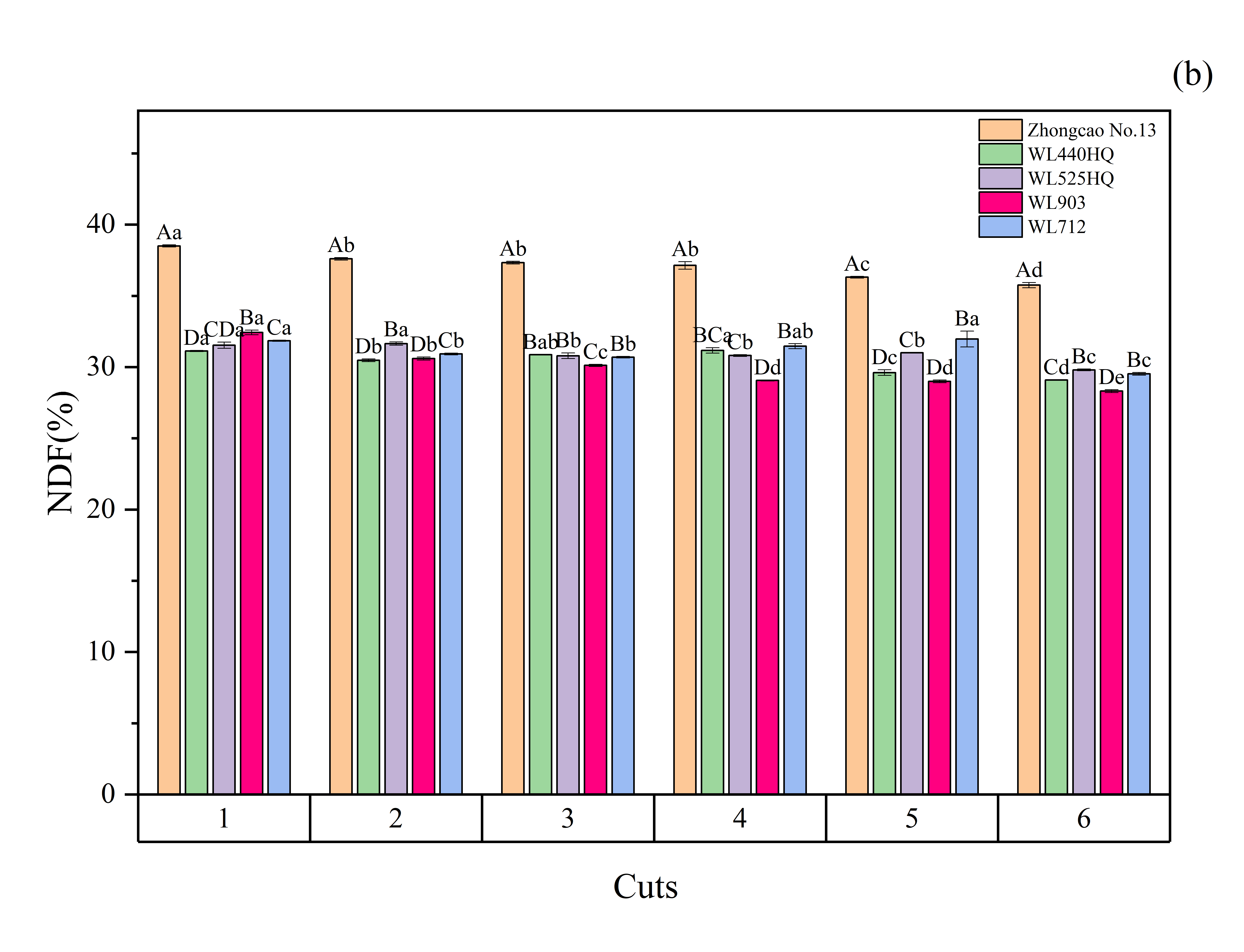

Supplement: S4 Fig — (TIF) [file pone.0346431.s005.tif]

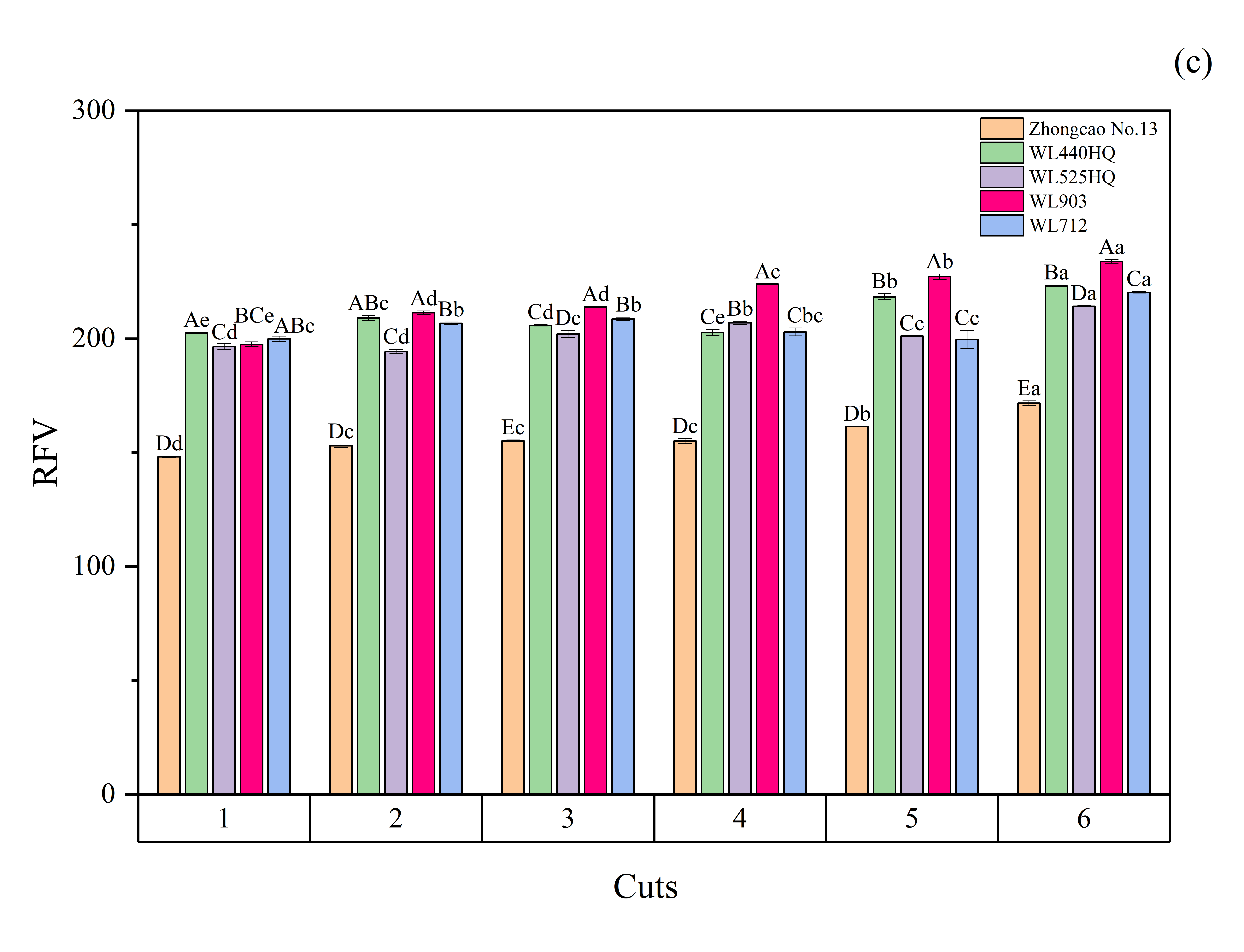

Supplement: S5 Fig — (TIF) [file pone.0346431.s006.tif]

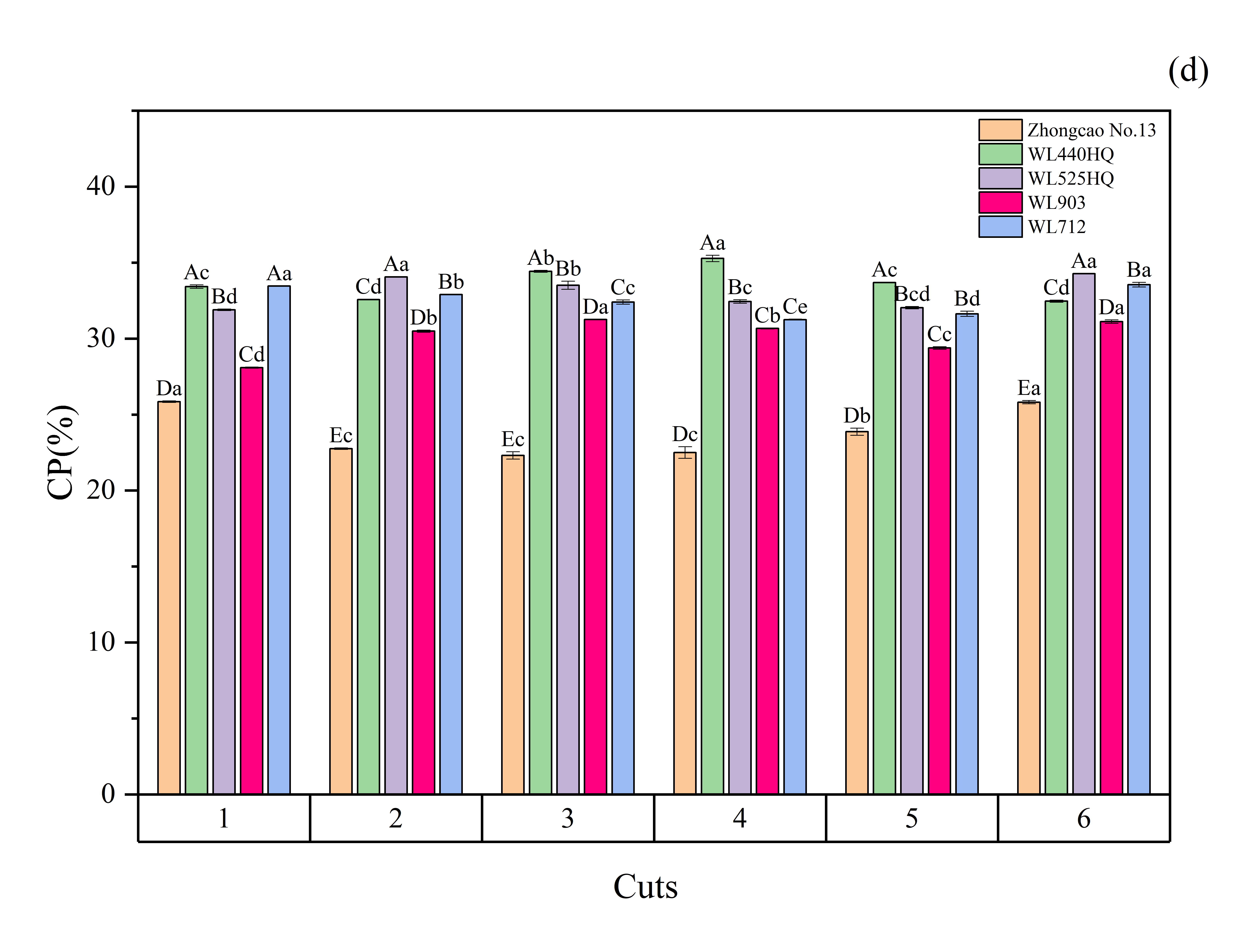

Supplement: S6 Fig — (TIF) [file pone.0346431.s007.tif]

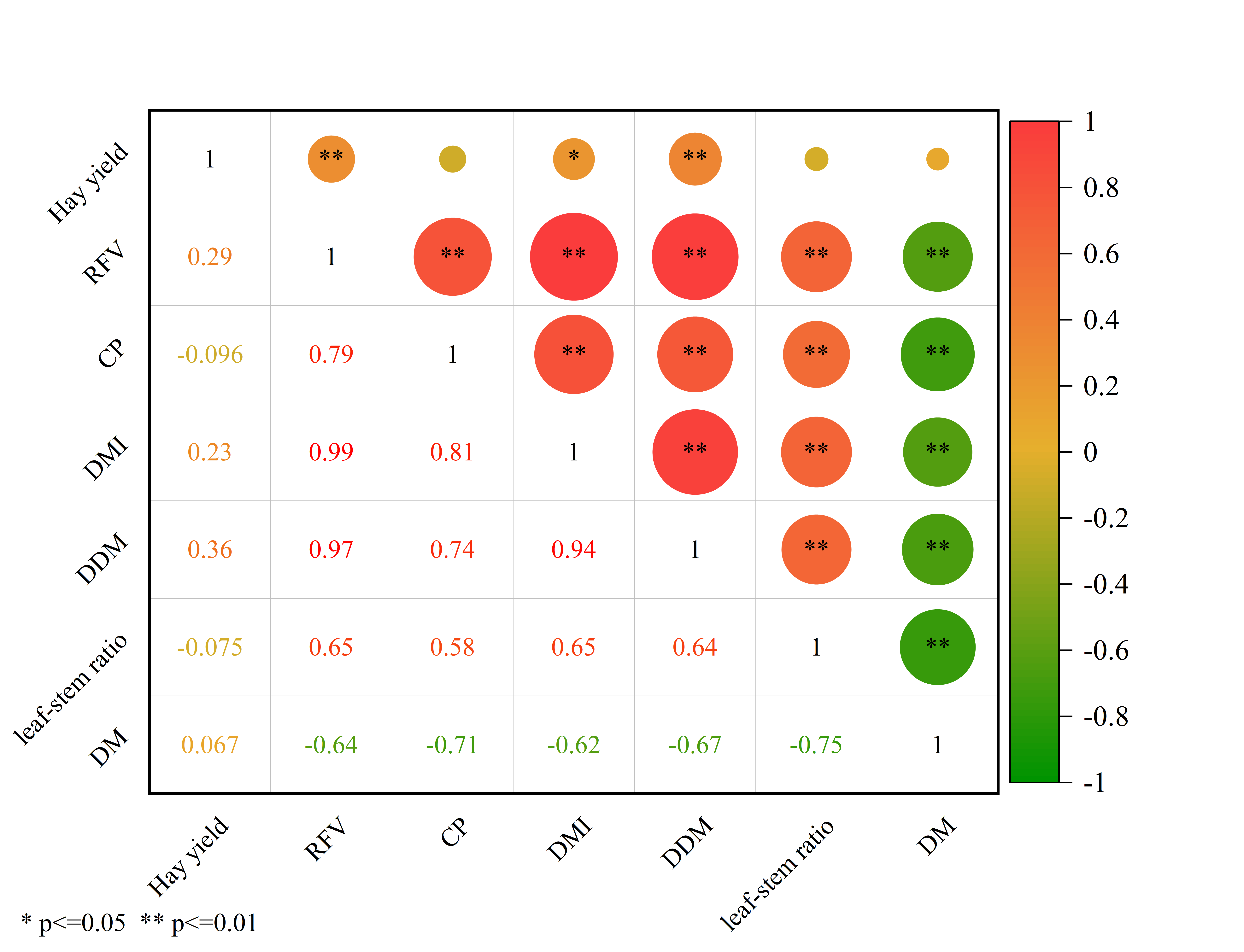

Supplement: S7 Fig — (TIF) [file pone.0346431.s008.tif]

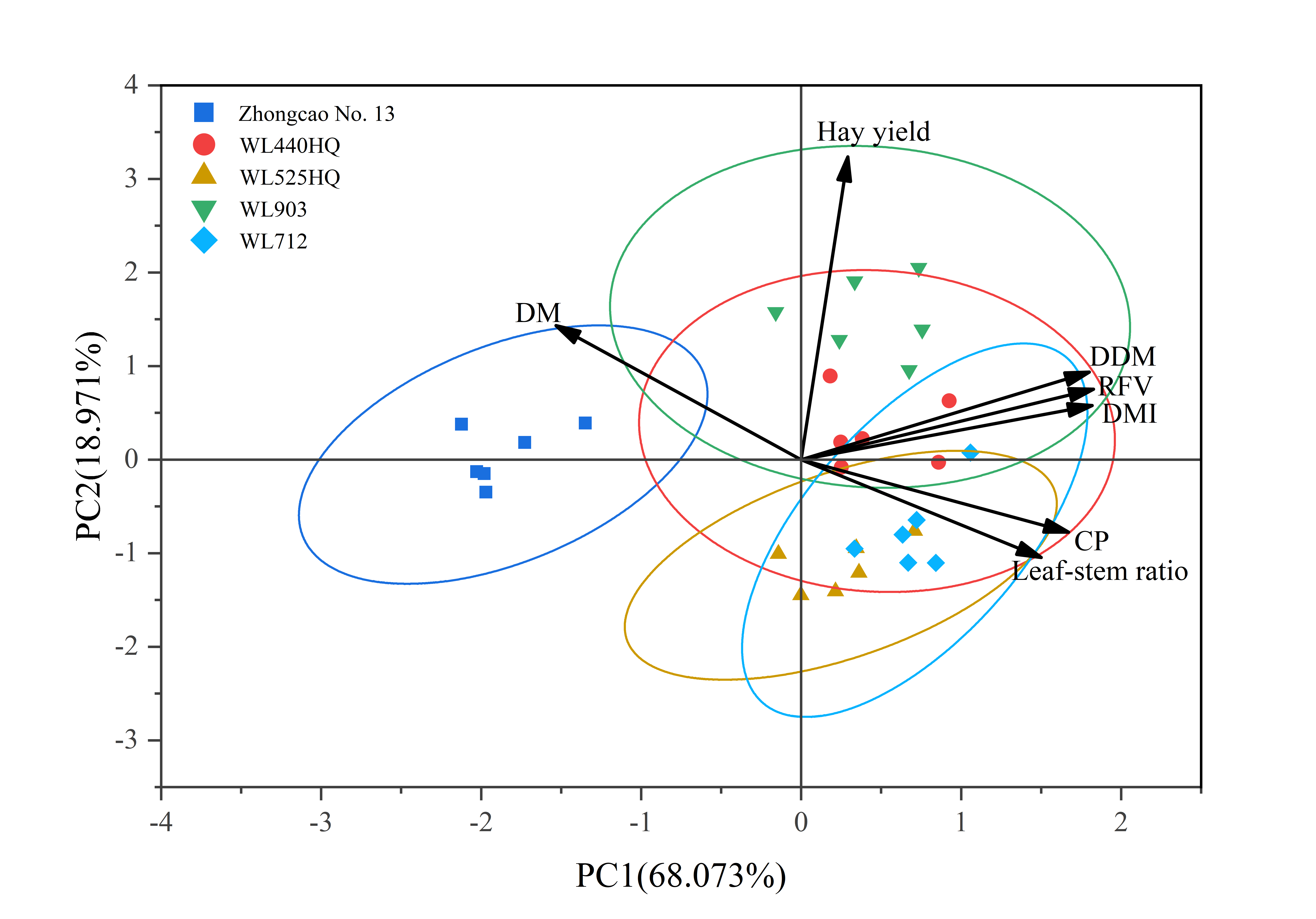

Supplement: S8 Fig — (TIF) [file pone.0346431.s009.tif]
