# Supplementary material for: Effects of harvest number on the yield and quality of different alfalfa varieties under hydroponic conditions
Source: PLoS One. 2026 Apr 16;21(4):e0346431. doi: 10.1371/journal.pone.0346431 (PMC13086426; doi:10.1371/journal.pone.0346431)
Supplement: Attachment — Submitted filename: Response_to_Reviewers_auresp_2.docx [file pone.0346431.s011.docx]

Response to Editor

Dear Debasis Mitra,

We sincerely appreciate your letter and the reviewers' insightful and constructive comments on our manuscript entitled “Effects of harvest number on the yield and quality of different alfalfa varieties under hydroponic conditions ” Their valuable input has significantly contributed to the refinement of our manuscript and offered critical guidance for our future research endeavors. Following a thorough analysis of the reviewers' feedback, we have meticulously prepared a comprehensive, point-by-point response.

1.We have meticulously revised our manuscript in preparation for resubmission.

2.We have thoroughly revised the manuscript in response to the reviewers' comments and have provided well-reasoned, point-by-point responses to each reviewer’s feedback.

3.All modifications in the revised manuscript have been highlighted in yellow, with precise references to the corresponding reviewer comments for ease of review.

We sincerely appreciate the opportunity to improve our manuscript and look forward to the possibility of its publication in PLOS ONE.

Best regards.

Response to reviews

Reviewer 1

Comment 1:The RFV unit is given in the table as a percentage. RFV is unitless.

Response:Thank you for your suggestion. I have removed the unit for RFV in the tables.

Comment 2:It is uncommon to see both "ABC" and "abc" lettering appearing together in tables. A change may be necessary at this point.

Response:Thank you very much for this detailed comment, which has led us to a clearer description of the statistical presentation of the table. The use of both lowercase and uppercase letters in our tables is not a format error, but a standard and necessary presentation determined by the repeated measures analysis of variance method used in this study. In this design, “variety” is an inter-group factor, and “harvest time” is an intra-group factor ( repeated measurement ). Therefore, data analysis will naturally produce two different types of post-hoc comparisons : 1.For intra-group factors ( harvest ) : We compared the differences in different harvest times ( harvest ) within the same variety. The lowercase letters ( a, b, c... ) in the table represent this vertical, time-series comparison. 2.For between-group factors ( varieties ) : We compared the differences between different varieties under a given stubble. The uppercase letters ( A, B, C... ) in the table represent this horizontal, treatment-group comparison. Nevertheless, we fully understand your original intention. In order to make readers understand the table more clearly, the analysis method has been explained in the notes.

Comment 3:The summary could be further detailed.

Response:Your point is indeed crucial. I have revised the abstract section accordingly, providing a more detailed description of the experimental content and results.

Comment 4:Based on which parameter did you select five varieties of alfa-alfa? i.e yield or production, quality, local variety, resistance? Please describe it with a sentence to the experimental design paragraph.
Did you follow a protocol to establish 10 h light for the experiment? Please insert a reference or if this procedure is yours, please indicate within the text.
Fig 1, Fig 2 and Fig 3 please make them larger in order to read data inside the graphs.
Please describe which were principal components that you have analyzed? i.e yield, quality, production

Response:Thank you for your very useful comments. I have explained the reasons for choosing these five alfalfa varieties in the experimental design section and explained the origin of the lighting time design. I have enlarged the picture. In the results and discussion section of principal component analysis, the specific analysis of which indicators is described in detail.
